# Does vitamin D affect strength and speed characteristics and testosterone concentration in elite young track and field athletes in the North European summer?

**DOI:** 10.1186/s12937-023-00848-7

**Published:** 2023-03-08

**Authors:** Eduard Bezuglov, Maria Shoshorina, Artemii Lazarev, Anton Emanov, Egana Koroleva, Ilsyuyar Anishchenko, Zbigniew Waśkiewicz‬, Mikhail Butovskiy, Ryland Morgans

**Affiliations:** 1 Department of Sports Medicine and Medical Rehabilitation, Sechenov First Moscow State Medical University, Moscow, Russia; 2 Sirius University of Science and Technology, Sochi, Russia; 3 High Performance Sport Laboratory, Moscow Witte University, Moscow, Russia; 4 Academy of Talents, Moscow, Russia; 5 Department of Internal Medicine, Mount Sinai Hospital, Chicago, IL USA; 6 Smart Recovery Sports Medicine Clinic, Moscow, Russia; 7 Central Clinical Hospital of the Administrative Directorate of the President of the Russian Federation, Moscow, Russia; 8 Institute of Sport Science, Jerzy Kukuczka Academy of Physical Education in Katowice, Katowice, Poland; 9 FC Rubin, Kazan, Russia

**Keywords:** Athletics, Vitamin D, Testosterone, Strength, Speed

## Abstract

**Background:**

Currently there are no data examining the relationship between the serum concentration of vitamin D bio-chemical marker 25(OH)D and strength and speed characteristics in elite young track and field athletes. Moreover, there are currently no data examining the correlation of vitamin D status with testosterone concentration in elite young track and field athletes. In studies involving members of the general population and athletes from other sports, conflicting data have been reported.

**Material and methods:**

Athletes (*n* = 68) from both genders took part in this study. Male athletes (*n* = 23) with mean ± SD age of 18.2 ± 1.9 years and female athletes (*n* = 45) with mean ± SD age of 17.3 ± 2.6 years participated. All athletes were ranked in the Top-3 in their respective age group and their corresponding results were listed in the Top-20 European records according to https://www.tilastopaja.eu/ in 2021.

**Results:**

The average 25(OH)D concentration was 36.5 ± 10.8 ng/mL and 37.8 ± 14.5 ng/mL in male and female athletes respectively. The prevalence of 25(OH)D deficiency (below 20 ng/ml) in both genders was only 5.8%. In the whole group, only 27.9% of athletes had 25(OH)D concentrations between 20 and 30 ng/ml, while 66.2% of athletes had concentrations above 30 ng/ml. There was no difference in vitamin D status between male and female athletes. There was no statistically significant Kruskal-Wallace test correlation between 25(OH)D concentration and performance in the 20 m and 30 m sprint, counter-movement jump and broad jump. There was no correlation between serum concentrations of 25(OH)D and total testosterone in either male or female athletes.

**Conclusion:**

In elite young track and field athletes who permanently live and train in an area above 50° north latitude, the prevalence of vitamin D deficiency in the summer months was much lower than in previously published studies examining an athletic population, that may be related to the training process. In this specific group of athletes, no correlation was found between serum 25 (OH) D concentration and strength and speed characteristics or total testosterone concentration.

## Introduction

Over the last few decades, scientists and practitioners have focused their interest on studying the possible effects of vitamin status and vitamin consumption on various aspects of athletic performance. Previous research has focused on the effects of vitamin D on the success and well-being of athletes and physically active members of the general population [1]. Vitamin D is widely considered important for bone health and skeletal muscle growth, and for immune and cardiopulmonary system function, inflammation modulation, recovery from injuries and reducing the risk of infection[2, 3]. The significance of the potential problem is highlighted by the pre-disposition of different sporting athletes to vitamin D deficiency, especially during the winter months. The high prevalence of vitamin D deficiency has been described in young and adult professional athletes living in regions with low (Russia, Poland, Ireland) and high insolation levels (Spain, Qatar, Croatia, Australia) [4–7]. The existing published data highlights that the main reason for vitamin D deficiency in athletes is residing in areas above 40° north latitude, especially during the winter months, when wearing clothing that covers much of the body surface and training indoors occurs [8–11]. This is partly due to the synthesis of this vitamin in the human body after skin exposure to sunlight containing ultraviolet radiation, which is the main source of vitamin D in its natural form. This pathway is the main source of vitamin D generation in the human body. This vitamin can also be orally ingested through food, such as cod liver oil, rainbow trout, salmon and raw mushrooms, although it requires the consumption of large quantities that is practically very difficult to achieve [12, 13].

An acceptable method to maintain appropriate vitamin D status is to ensure outdoor activity with high levels of insolation, to achieve sun exposure for 5–30 min at least twice a week ensuring the face, arms and legs are revealed [14]. However, even athletes who regularly exercise outdoors for extended periods of time during the summer months may still have insufficient 25(OH)D concentration, which may partly be attributed to exercise-induced stress [15–17]. Thus, the positive effects of vitamin D supplementation on various organ and system functions have been previously proven, although conflicting data are currently available regarding its effect on skeletal muscle function. The mechanism by which vitamin D provides these beneficial effects in skeletal muscle has not been definitively established but has been extensive investigated. Recent data suggest that vitamin D function on skeletal muscle occur via a direct mechanism, namely the receptor for 1.25-dihydroxyvitamin-D3 (vitamin D receptor (VDR)) [18]. The positive role of vitamin D on skeletal muscle physiology can also be partly explained by the activation of gene expression affecting muscle growth and differentiation, especially in type II fast-twitch fibres [19–23]. More recently, the positive effect of vitamin D on muscle protein synthesis, adenosine triphosphate concentration, strength, speed, power, jump height and the ability to perform aerobic and anaerobic exercise have been demonstrated [2]. The crucial importance of vitamin D for muscle performance may be due to both its genomic and non-genomic effects, affecting muscle calcium and phosphate transport through cell membranes, phospholipid metabolism, muscle cell proliferation and differentiation [23].

The relationship between concentrations of the vitamin D bio-chemical marker 25(OH)D and total testosterone has also been extensively studied. This interest is easily explained, as high levels of testosterone can lead to strength and endurance advantages in many sports. The assumption that there may be a relationship between the concentrations of these two biological agents is based on the fact that receptors for vitamin D are also found in tissue associated with testosterone production [24]. This, combined with the observed correlation between vitamin D levels and testosterone in the general population, suggests that vitamin D may also influence testosterone concentration in athletes [25, 26]. A possible mechanism for this effect may be based on the fact that the vitamin D receptor (VDR), which mediates the biologic effect of vitamin D, is expressed in tissue throughout the male reproductive tract, including Leydig cells [27]. Furthermore, there is evidence to suggest that the development of hypogonadism in the absence of VDR activity [28] and the tissue-specific effect of vitamin D on estrogen and androgen production and metabolism accounts for a significant increase in testosterone production [29]. These mechanisms may influence gonadal function in both sexes which may include the effect of calcium homeostasis on estrogen bio-synthesis or direct regulation of aromatase gene expression [28]. However, the exact mechanisms that could convincingly demonstrate a potential association between these parameters are currently unknown, and the results of studies in athletes are contradictory. For example, no correlation between vitamin D status and total testosterone was found in Canadian junior ice hockey players or in power measures in Polish track and field athletes [30, 31]. However, in another study examining Italian soccer players, such a correlation was reported [32].

Recent evidence stated that vitamin D deficiency can cause strength deficits and lead to degeneration of type II muscle fibres, which have been found to negatively correlate with physical performance [33]. While another review reported that vitamin D supplements improved vitamin D status and had a positive effect on skeletal muscle in both athletes and the general population [34]. Additionally, sport-specific research also reported a correlation between serum 25(OH)D concentration and speed and power performance in young soccer players [6] and muscle strength, speed and endurance in adult professional soccer players [35]. However, in contrast, meta-analyses conducted found no evidence of a positive effect of vitamin D supplementation on physical performance and muscle strength [36, 37]. More specifically, Kim et al. also highlighted that vitamin D deficiency did not correlate with shoulder muscle strength in professional volleyball players [38], while Bezuglov et al. also stated that no correlation was found between serum 25(OH)D concentration, muscle strength and running speed in young professional soccer players [5].

Thus, evidently, there is currently conflicting research on the effects of vitamin D on various measures of physical performance. However, most studies have investigated the effect of vitamin D supplementation and not vitamin D status on physical performance. At present, there are only limited data available regarding the effect of vitamin D status on physical performance in elite track and field athletes, and to our knowledge, no previous study assessed the effects of permanently residing above 50° north latitude. This study would provide novel insights into this topic and contribute to understanding the correlation between vitamin D status and physical performance in elite young athletes. Moreover, there are currently only sporadic studies examining the correlation between vitamin D status and testosterone concentration in elite young track and field athletes. Therefore, the aim of this study was to investigate the prevalence of vitamin D deficiency and to assess the effects of vitamin D status on strength and speed characteristics and testosterone concentrations in elite young track and field athletes who permanently reside in areas above 50° north latitude.

## Hypothesis

Three hypotheses were tested in this study:

1. The prevalence of vitamin D deficiency in elite young track and field athletes will be low during the summer months, due to the greater time of outdoor training and the elite young track and field athletes' clothing (more exposed body-parts).
2. The serum 25(OH)D concentration will not be associated with the concentration of total testosterone in a group of elite young track and field athletes.
3. Serum 25(OH)D concentrations have no relationship with strength and speed characteristics in elite young track and field athletes of either gender.

## Methods

### Subjects

The current investigation collated data from a cohort of 68 white athletes from an elite European “Academy of Talents” (See Table 1 for participant information mean ± SD). The study was conducted in August 2021 before the start of the training camp. All subjects had no medical contraindications to perform athletics and had specialized in athletics for between 3–13 years. All athletes were selected by an expert council, consisting of five elite coaches with at least 20 years of experience, to participate in camps for the most talented athletes in their respective age group in Russia. All athletes at the time of participation were ranked in the Top-3 in their age group and their corresponding results were listed in the Top-20 European records according to the https://www.tilastopaja.eu/ website in 2021. All athletes specialized in the short sprint (100 m and 200 m, 110 m hurdles), 400 m and 400 m hurdles, long jump (single and triple), high jump or throwing disciplines. The study was performed in accordance with the Helsinki Declaration principles and was approved by the Local Ethics Committee of the Sechenov First Moscow State University under statement number 11–19 of 07/25/2019. All subjects provided their written informed consent. Informed written consent was provided by the parents of subjects under 18 years of age. Athletes who were 18 years or older provided their own written consent. Subjects were fully familiarized with the experimental procedures within this study due to the regular testing protocols implemented as part of the Academy performance monitoring strategy.

**Table 1:** Characteristics of male and female track and field athletes by age, height, weight, BMI and vitamin D status

	**Male (mean ± SD; min, max) *n* = 23**	**Female (mean ± SD; min, max) *n* = 45**	***P* value**
Age, years	18.2 ± 1.9 (15.5, 23.1)	17.3 ± 2.6 (12.8, 23.2)	0.15
Height, cm	185 ± 7.3 (166, 198)	171 ± 6.0 (162, 184)	< 0.001
Weight, kg	74.5 ± 10.5 (53.9, 99.0)	61.6 ± 16.5 (36.3, 95.0)	< 0.023
BMI	21.8 ± 2.5 (18.8, 31.2)	20.3 ± 2.5 (16.6, 30.1)	0.002
25(OH)D, ng/ml	36.5 ± 10.8 (20.1, 63.2)	37.8 ± 14.5 (18.6, 82.8)	0.71

All athletes permanently resided in areas between 50° and 57° north latitude. The inclusion criteria for the study included: participated in athletics for at least three years; permanently reside and train in Russia; no injuries resulting in missing more than three training sessions in the last 30 days prior to the study; and a prize-winning place in the national championship 12 months before participation in the training camp. The exclusion criteria for the study included: athlete had consumed vitamin D supplements seven days or less prior to the blood test; athlete had used sunscreen during outdoor training sessions; athlete had spent more than seven days outside Russia in the last three months; athlete has missed more than three days of training due to injury in the last 30 days prior to the study; and athlete had consumed dietary supplements seven days or less before blood testing.

### Laboratory tests

All subjects had a single blood sample taken in the morning. Blood sampling was carried out in one day in August 2021 between 8:00 and 10:00 a.m. from the cubital vein by an experienced technician. All athletes were instructed to rest on the day prior to blood sampling and refrain from alcohol consumption. Analysis of total 25-hydroxyvitamin D (25-OHVITD) in the blood were performed by liquid chromatography-mass spectrometry (LC–MS) on an Agilent 1200 liquid chromatography (Agilent, USA) combined with an AB Sciex 3200 MD mass detector (Sciex, USA). 25 (OH) D concentration was measured using total 25-hydroxyvitamin D levels (25-OHVITD), which is currently considered the most appropriate to reflect vitamin D stores in the body [39, 40]. Vitamin D bio-chemical marker 25 (OH) D values above 30 ng/ml were considered normal, 20-30 ng/ml were considered insufficient, and below 20 ng/ml were considered deficient [14]. Analysis of total testosterone in the blood were performed by liquid chromatography-mass spectrometry (LC–MS) on an Agilent 1200 liquid chromatography (Agilent, USA) combined with an AB Sciex 3200 MD mass detector (Sciex, USA). The separation of substances was conducted in a gradient mode, where an acetate buffer was used during the aqueous phase, and methanol was utilized during the organic phase. A reverse-phase column (Phenomenex, USA) was used during the stationary phase. All reagents used were labeled no lower than HPLC-grad, considering the standards for substances produced by TRC (Canada). These procedures have been previously validated and are based on published guidelines for obtaining the most valid, reliable and accurate testosterone values [41, 42].

### Power and speed testing

After the standardized warm-up routine, subjects performed two 20 m and two 30 m sprint trials on an official running track surface (Regopul®, UK). Between the sprint efforts a 3-min recovery period was provided. The best single effort from the sprints was used for analysis. Sprint times were recorded using SmartSpeed® Pro timing lights (Fusion Sport®, Coopers Plains, Australia), with gates at 0 m, 20 m and 30 m. This system uses a single-beam design to improve battery life and ease of set-up, and also incorporates novel error detection algorithms to reduce false triggers. In the event of multiple triggers, the algorithm interprets the longest trigger as the true start time. Gates were set at a height of 1 m from the floor. Each attempt was recorded with an accuracy of one hundredth of a second. The SmartSpeed® Pro timing system has previously been validated and used to evaluate sprint performances in male students and recreational female athletes [43, 44]. Subjects started all sprint trials from a two-point start position, with their front foot 0.3 m behind the first timing gate, and were instructed to complete with maximum effort. All tests were carried out in specific athletic shoes regularly worn by the subjects. All subjects were familiar with the sprint test protocols, having completed several practice testing sessions.

Following the 3-min recovery after the final sprint attempt, counter-movement jumps (CMJ) without arm-swing were performed. The arms were fixed. All subjects were familiar with the jumping protocols, having completed jumps regularly as part of the academy assessment procedures and participating in several practice testing sessions. In an attempt to standardize jump tests, subjects were instructed to perform all attempts in accordance with the protocols outlined by Cormack et al. [45]. For each jump test, three attempts were performed and the best result was recorded in centimeters (cm) for further analysis. A recovery interval of 3-min between jumps was provided. A commercially available jump mat (Vald Performance™, FusionSport, Australia) was used which has been previously validated [46, 47].

Following the 3-min recovery after the final CMJ attempt, Broad Jump (BJ) landing on both feet without moving were performed. All subjects were familiar with the jumping protocols, having completed jumps regularly as part of the academy assessment procedures and participating in several practice testing sessions. For each jump test, three attempts were performed and the best result was recorded in centimeters (cm) for further analysis. A recovery interval of 3-min between jumps was provided.

All jump tests were conducted in an indoor facility to avoid any external variations in surface that might affect results. Subjects performed the tests in their normal sports shoes, in an indoor facility with a non-slip, flat surface at a room temperature of 18–20 C°.

### Body composition

Musculoskeletal mass was assessed using bio-impedance analysis on the day following the blood sampling procedure. The ABC-02 “MEDASS” (Russia) analyzer was used for bio-impedance analysis. The parameters in the analysis were calculated using previously validated equations [48]. Body height and weight measurements were obtained from all subjects. The musculoskeletal mass analysis was conducted in the morning on an empty stomach using a single measurement methodology. During the test days, the female subjects were not in their menstruation cycle. In all athletes’ muscle, body fat mass (kg) and lean weight (kg) were assessed.

### Skill level

All athletes were divided into several groups, depending on skill level. This selection was conducted according to the classification adopted in Russia, where the highest rank is held by “honored masters of sports” (most often these are Olympic medalists and World Champions), "international-class masters of sports" (most often participants in the Olympic Games, winners of the World and European championships), "masters of sports" (most often champions and prize-winners of Russian championships at different age groups), and "candidates for master of sports" (most often participants in the finals of Russian championships). In previous years, young athletes who have the “first-class” and “second-class” rank have often been the winners of the adult Russian regional championships.

### Statistical analysis

Data was stored in MS Excel and analysis was performed using IBM SPSS 26.0 (Armonk, USA). Normality of the quantitative data was analyzed using Shapiro–Wilk test. A two-sample independent T-test for unequal variances was used to assess the inter-group differences (age, height, weight and BMI) in case of normal distribution. The Mann–Whitney U-test was used to assess the significance of inter-group differences for playing minutes distributed non-normally. Kruskal–Wallis test was used to assess the inter-group differences in vitamin D status. Multiple regression was used to assess the relationship of any quantitative indicators. Categorical data (vitamin D status) was described using frequency charts showing absolute values and its percentages. Chi-squared test was used to estimate any differences in vitamin D status in male and female athletes in 2 × 4 tables. Values at *p* < 0.05 were considered statistically significant.

## Results

When dividing the athlete sample according to gender (male and female) no differences by age (*p* = 0.15) were reported, although male athletes were significantly taller and heavier (*p* < 0.001). Body Mass Index was also significantly higher in men (*p* = 0.023). The concentration of the vitamin D bio-chemical marker 25(OH)D was 36.5 ± 10.8 ng/ml and 37.8 ± 14.5 ng/ml in male and female athletes respectively (*p* = 0.71) (Table 1).

In the female athletes, age significantly correlated with height (*r* = 0.35, *p* = 0.014), weight (*r* = 0.53, *p* < 0.001) and BMI (*r *= 0.46, *p* = 0.001), but did not significantly correlate with vitamin D status (*r* = 0.21, *p* = 0.15). In the male athletes, age close to statistical significance was correlated with height (*r* = 0.38, *p* = 0.05) and weight (*r* = 0.39, *p* = 0.05), but did not correlate with vitamin D status (*r* = 0.17, *p* = 0.39) or BMI (*r* = 0.19, *p* = 0.36).

All athletes were also divided into three groups depending on 25 (OH) D concentration in the blood; deficient, insufficient and normal (Table 2). The prevalence of vitamin D deficiency (below 20 ng/ml) and insufficiency (20-30 ng/ml) in all athletes was 5.9% and 27.9% respectively, while 66.2% reported normal vitamin D levels (above 30 ng/ml). There was no difference between the prevalence of different vitamin D status between male and female athletes (X = 2.96, *p* = 0.40).

**Table 2:** Vitamin D status in athletes

Vitamin D	All n (%)	Male n (%)	Female n (%)
Deficient (below 20 ng/ml)	4 (5.9%)	2 (8.7%)	2 (4.4%)
Insufficient (20-30 ng/ml)	19 (27.9%)	4 (17.4%)	15 (33.3%)
Normal (above 30 ng/ml)	45 (66.2%)	17 (73.3%)	28 (62.3%)

When examining the dependence of strength, speed and body composition, no statistical significance in parameters such as 20 m and 30 m sprint, CMJ and BJ on vitamin D status according to the Kruskal-Wallace criteria were found (Table 3).

**Table 3:** Dependence of speed and power indicators on the vitamin D status

Parameter	*p* value
20 m	0.72
30 m	0.64
Counter-movement Jump	0.25
Broad Jump	0.80

No significant association between 25 (OH) D concentration and the analyzed parameters of strength, speed and body composition were found in age- and gender-adjusted male and female athletes, and independently in age- and gender-adjusted male and female athletes (Table 4).

**Table 4:** The association between 25(OH)D concentration and various analyzed parameters of strength, speed and body composition. Both genders are gender-adjusted, males and females are age-adjusted

Parameter	All	Male	Female
Fat mass	0.41	0.65	0.88
Muscle mass	0.64	0.65	0.43
Broad jump	0.82	0.93	0.32
30 m	0.27	0.55	0.09
20 m	0.19	0.52	0.07
Counter-movement jump	0.23	0.35	0.33

**Fig. 1:**
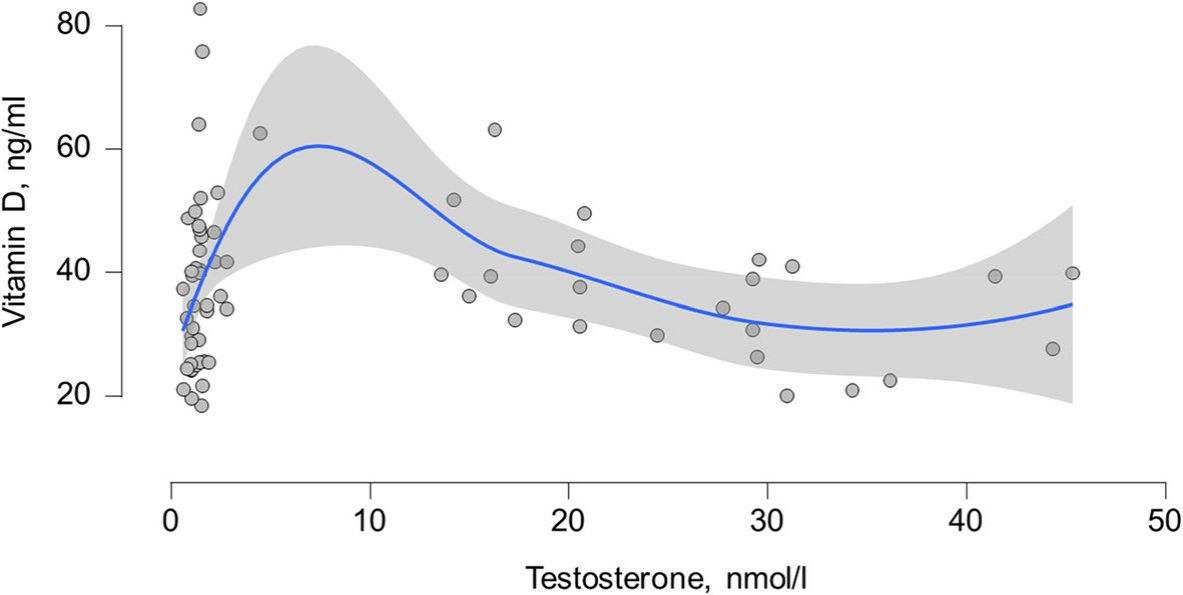
Correlation between 25(OH)D concentration and testosterone concentration in all subjects

**Fig. 2:**
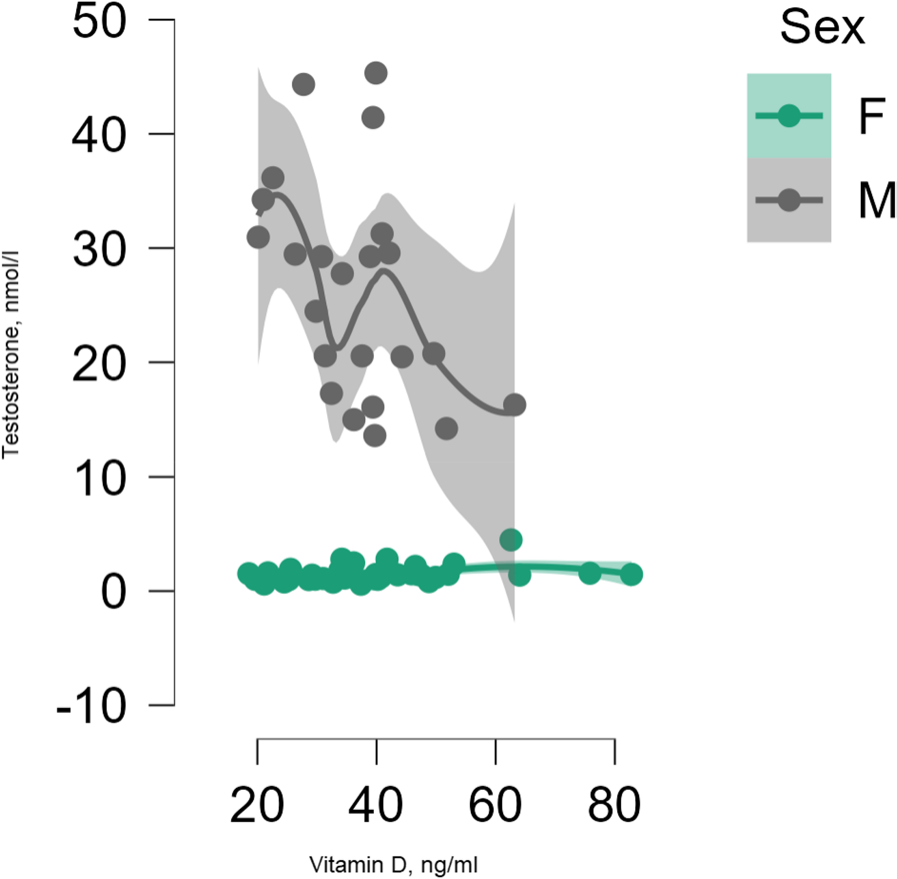
Correlation between 25(OH)D concentration and testosterone concentration among male and female track and field athletes, adjusted for age and body mass index

When analyzing the vitamin D bio-chemical marker concentration and testosterone in all subjects, no significant correlation was found (*r* = 0.09, *p* = 0.43) (Fig. 1). When considering gender (*p* = 0.18) and when adjusted for age and BMI (*p* = 0.10), the association between these parameters was also statistically in-significant (Fig. 2).

The age of both male and female athletes did not significantly correlate with any of the strength and speed measures and serum 25(OH)D concentrations (*p* = 0.35 and *p = *0.89 respectively). The vitamin D bio-chemical marker 25(OH)D concentration was not associated with the skill proficiency of either male (*p* = 0.70) or female (*p* = 0.71) athletes.

## Discussion

The study aimed to examine the prevalence of vitamin D deficiency and the relationship between the concentration of vitamin D bio-chemical marker 25(OH)D and various characteristics of strength and speed in elite young track and field athletes. Furthermore, to investigate any correlation between vitamin D bio-chemical marker 25(OH)D and testosterone concentration in elite young track and field athletes. Collectively, the data show that the study hypotheses were confirmed. In this study it was shown that the majority of elite young track and field athletes’ that reside and train in areas between 50° and 57° north latitude have no vitamin D deficiency during the summer months. Concurrently, the average concentration values are close to the lower limit of normal, possibly suggesting the need to monitor vitamin D bio-chemical marker 25(OH)D during the autumn and winter months, when a significant part of training occurs indoors and the level of sun exposure is significantly reduced.

In this study, the level of vitamin D insufficiency detected was lower than in previous studies in both athletes and the general population. An analysis of 14 population-based studies with a sample of 55,844 European participants showed that regardless of age, ethnicity or geographical location, the prevalence of vitamin D deficiency (below 20 ng/ml) was 40.4% [49]. While a systematic review reported that more than 37% of the world's population have circulating 25(OH)D concentrations below 20 ng/ml [50]. Studies examining athletes have also confirmed the high prevalence of vitamin D deficiency in serum, where it was found that 64–83% of English, Spanish and Polish soccer players were deficient [16, 17, 51]. Furthermore, a very high prevalence (84%) of vitamin D deficiency or insufficiency was reported in 342 Qatari soccer players that resided in the Middle East (Qatar) [52].

The factors possibly affecting vitamin D status are exercise stress and exercise itself. In a study by Andersen et al. investigating female military personnel it was shown that exercise stress can play a regulatory role in vitamin D levels and reduce vitamin D concentrations even during prolonged outdoor exercise in summer and early autumn months [15]. Similar results have been obtained in more recent studies involving Polish and English adult professional soccer players who trained outdoors during the summer months, [16, 17]. Thus, exercise-induced stress may affect vitamin D status in athletes, which may possibly be related to the immune-suppressive effect of intense stress [53]. Although, exercise itself can also increase vitamin D levels. This has been found in some observational studies, which showed that regular exercise or high physical activity was associated with higher serum 25(OH)D concentrations, even after adjusting for sun exposure [54–56].

One of the most notable factors contributing to vitamin D deficiency in athletes is residing in regions north of the 35^th^ northern latitude, such as Russia and Scandinavian countries. This is due to the greater angle at which the sun's rays enter the atmosphere in these regions, resulting in scattering [11]. This concept is supported by research examining Finnish runners and gymnasts living at 60° north latitude where deficiencies in serum 25(OH)D concentration were found in over 80% of this subject population [57]. While in another study conducted during the winter months, examining elite young soccer players aged 16 years who permanently reside in Moscow, vitamin D deficiency was found in more than 40% of participants [5]. In our study, however, serum 25(OH)D concentration below 20 ng/ml was only found in 5.8% of participants. It is possible that the main reason for this low prevalence of the vitamin D insufficiency was the training process of the track and field athletes. During the summer months, all athletic training occurs outdoors, where these training sessions are often performed for at least two hours in duration, 5–6 times a week, and training consistently commenced at 9.00 a.m. Furthermore, the clothing worn by these athletes was also important to ensure legs, arms, neck and face were exposed to UV rays during training. It should also be noted that none of the study subjects administered sunscreen or head protection during training. Therefore, it may be considered that remaining outdoors for at least two hours, 5–6 times per week during the summer months in geographical regions above 50° north latitude maintains acceptable serum concentration of the vitamin D bio-chemical marker 25(OH)D. However, the average value of the serum concentration was close to the lower limit of normal, which possibly warrants the necessity to continuously monitor vitamin D bio-chemical marker 25(OH)D during the autumn and winter months.

It has previously been hypothesized that vitamin D may indirectly increase testosterone production and thus positively affect the muscular system [58]. In our findings no significant correlation between serum 25 (OH) D concentration and total testosterone in either males or females was reported. However, in previous studies, this relationship has only been found in the general population with some pathology. In a study examining the elderly population with obesity and a baseline of relatively low total testosterone, testosterone concentration was shown to increase with vitamin D supplementation [58]. While another study examining members of the general population aged 18–60 years demonstrated a direct and positive relationship between serum vitamin D level and overall semen quality, male reproductive potential, and testosterone concentration in a group with seminal abnormalities [59]. However, research investigating the association between serum 25(OH)D concentration and total testosterone in healthy members of the general population have not been confirmed. The Lerchbaum et al. study found no evidence a vitamin D supplementation effect on total testosterone concentration in healthy middle-aged men with normal baseline testosterone concentration [60]. Furthermore, Książek et al. reported no significant differences were found between the average total testosterone and low serum 25(OH)D concentrations in 176 healthy young, active men aged 18–35 years from a genetically homogeneous population in Lower Silesia (Poland) [61]. Thus, it may be argued that the currently available data on the possible relationship between the vitamin D bio-chemical marker 25(OH)D and total testosterone are inconsistent, and our study involving elite young track and field athletes, to our knowledge, is the first to do so. However, previous work has shown a correlation between serum 25(OH)D concentration and strength, speed and endurance performance in athletes, although in our work we found no such effect in elite young track and field athletes.

Some studies have shown that low 25(OH)D concentration can directly affect muscle strength and performance [62]. However, these studies were conducted in young and elderly people who do not participate in elite sport [21, 63, 64]. However, it should be noted that the positive effect of vitamin D on muscle function in athletes have been observed when any deficiency and insufficiency have been corrected by administering vitamin D3 supplements [65, 66]. Although, only athletes with initially low vitamin D levels had a positive effect on muscle strength and performance by increasing vitamin D concentrations [8]. While a systematic review found no effect of vitamin D supplementation and increased 25(OH)D concentration on various physical indicators, including muscle strength [36]. The descriptive review by Książek et al. also showed that previous studies were inconclusive and found no clear association between serum 25(OH)D concentration and performance [33]. Therefore, it may be argued that the available data on the effect of vitamin D on physical performance are in-consistent and limited to assessing the effect of supplementary vitamin D intake on muscle function in athletes with vitamin D deficiency or insufficiency. Studies investigating high-level athletes and the effect of actual vitamin D status on muscle function are currently scant. Although, in Greek adult soccer players a significant correlation between vitamin D status and squat jump, counter-movement jumps (CMJ), 10 m and 20 m sprint performance was obtained [35]. As in the studies conducted by Fitzgerald et al. and Krzywański et al., our research reported no relationship between serum 25(OH)D concentrations and total testosterone.

The limitations of our study include the lack of control for athlete sunlight exposure, such as standard erythemal dose calculations. Future studies should investigate the effects of vitamin D supplementation on strength and speed performance in elite athletes, as well as quantifying the effect of various training stress and exercise on vitamin D status. It is possible that increases in serum 25(OH)D concentration above a certain threshold may be associated with changes in various measures of physical performance. It may also be of real interest to assess the dynamics of serum 25(OH)D concentration during the competitive season and to develop interventions to correct any deficiencies or insufficiencies identified. Investigating the concentration of this vitamin in athletes and considering the specifics of their training and competitive activities would also be noteworthy. Finally, examining the possible association between vitamin D supplementation and high serum 25(OH)D concentration and the effect on total testosterone concentration may also be a worthwhile subject for future research.

## Conclusion

In elite young track and field athletes who permanently reside and train in an area above 50° north latitude, a low prevalence of the vitamin D deficiency was observed during the summer months, which may partly be related to the nature of their training process. In this group of athletes, no correlation was found between serum 25(OH)D concentration and total testosterone concentration with strength and speed characteristics .

## Data Availability

The datasets used and/or analyzed during the current study are available from the corresponding author on reasonable request.
